# How Efficient Is ChatGPT in Accessing Accurate and Quality Health-Related Information?

**DOI:** 10.7759/cureus.46662

**Published:** 2023-10-07

**Authors:** Ibrahim Ulusoy, Mehmet Yılmaz, Aybars Kıvrak

**Affiliations:** 1 Department of Orthopaedics and Traumatology, Selahaddin Eyyübi State Hospital, Diyarbakır, TUR; 2 Department of Orthopaedics and Traumatology, Gaziantep 25 Aralık State Hospital, Gaziantep, TUR; 3 Department of Orthopaedics and Traumatology, Avrupa Hospital, Adana, TUR

**Keywords:** health information, website, jama, discern, chatgpt

## Abstract

Background and objective

The field of artificial intelligence (AI) is advancing at a rapid pace, impacting all aspects of human life. Chat Generative Pre-trained Transformer (ChatGPT), which represents one of AI's most recent and remarkable achievements, has garnered significant attention and popularity in the academic community. ChatGPT, a language model-based chatbot developed by OpenAI, responds quickly and provides answers to the questions put to it. This chatbot has the ability to gather content from a variety of sources on the internet. However, its success in providing correct information has not yet been comprehensively analyzed. In light of this, this study aimed to engage in a comparative content analysis of health-related information provided by ChatGPT and a few selected websites.

Methods

We performed a qualitative analysis of data obtained from various information sources by using the DISCERN score and the Journal of the American Medical Association (JAMA) benchmark criteria. In addition, readability levels of the content were measured by using the Flesch-Kincaid grade level, Gunning Fog Index, and Simple Measure of Gobbledygook (SMOG) index.

Results

Based on our findings, there was no statistically significant difference between the websites and ChatGPT in DISCERN scores. However, the JAMA score was statistically significantly higher for websites. With regard to the Flesch-Kincaid grade level, Gunning Fog Index, and SMOG index values, the data obtained from the websites had higher readability.

Conclusion

Although AI is starting to play a significant role in our everyday lives, it has yet to surpass traditional methods of accessing information in terms of readability and reliability.

## Introduction

The concept of artificial intelligence (AI) refers to the ability of computers to simulate the capabilities of the human mind, i.e., to think, learn, make decisions, understand, and perform tasks in a human-like manner. AI combines a range of techniques and methods from computer science, statistics, mathematics, and other disciplines. AI is a technology with many different areas of application in the healthcare field. The healthcare sector can employ AI technology to monitor the health status of patients, accelerate the diagnosis process, and improve the treatment process. In addition, it can also be used to raise awareness and disseminate relevant information among patients.

Many patients may not have sufficient information about their diseases and treatment processes. In this context, AI technologies can improve the quality of health services by facilitating patients' access to information. For example, AI technologies can develop chatbots that can provide accurate and intelligible answers to patients' questions. These chatbots can answer patients' questions about their diseases, provide information about their treatment processes, and assist in accessing healthcare services. Chat Generative Pre-trained Transformer (ChatGPT; OpenAI, San Francisco, CA), an AI model equipped with natural language processing abilities, is such a chatbot.

There is a lot of information about diseases and health on the internet. People need to access this information when they need it. However, in today's crowded and chaotic digital environment, which is accessible to everyone, information pollution and misinformation are major concerns. In our study, we offer a comparative analysis of health-related information gathered using ChatGPT and that provided by a few health-related websites in terms of quality, reliability, and readability.

## Materials and methods

Internet searches to gather data for our study were conducted on February 12, 2023, on a Chrome browser. We cleared browser and cookie information prior to conducting the search. In our study, low back pain, meniscal injury, and gonarthrosis, which are some of the most common orthopedic disorders, were used separately as search terms [1-3]. ChatGPT (the version updated on August 3), an AI model, was asked questions related to these search terms (e.g., can you give information about low back pain/meniscal injury/gonarthrosis?). This chatbot, equipped with a "regenerate response" option, provided five different answers, which were documented. On the other hand, popular search engines such as Google (www.oogle.com), Yahoo (www.yahoo.com), Bing (www.bing.com), and Ask (www.ask.com) were used for browsing websites [4].

In line with previous similar studies, the top 10 sites appearing on the search engine were examined [5-7]. Generally, the most frequently viewed sites by internet users appear on the first page of search engines [6]. All inquiries in our study were made in English. Inaccessible and sponsored links, non-English content, advertisements, social media websites, and video-sharing and duplicate sites were excluded from the study. The assessment was performed by two doctors with at least five years of experience in orthopedic surgery. In cases of disagreement, consensus was reached by evaluating the test data together, and a data set was obtained for each test.

The quality of the information obtained was measured by using the DISCERN score and the Journal of the American Medical Association (JAMA) benchmark criteria, similar to previous studies [4,6,8-11]. DISCERN score, developed by Charnock et al. [12], is used by patients and information providers to evaluate the quality of health information. The DISCERN evaluation table consists of 16 questions in total. Each answer is given a score ranging from 1 to 5, and the cumulative DISCERN score obtained ranges from 16 to 80 [13]. Silberg et al. have advocated the use of JAMA criteria to evaluate the quality of information obtained from health-related information sources [13]. With these criteria, four basic features (authorship, attribution, disclosure, and currency) associated with health information resources/websites are evaluated. While performing the evaluation, care should be taken to clearly spell out each criterion. The scores obtained by the evaluation of four features range from 0 to 4. Each website is examined one by one, and a score of "1" is given to each item that meets these criteria [13].

As in previous studies, the Flesch-Kincaid grade level, Gunning Fog Index, and Simple Measure of Gobbledygook (SMOG) index were used to measure the readability of the information obtained [10,14]. The Flesch-Kincaid grade level is a readability test that is commonly used to assess the difficulty of a written text. It uses two factors to determine the grade level of a text: the average number of syllables per word and the average number of words per sentence. It is expressed in terms of a grade level, such as "Grade 6" or "Grade 12", which means that the text can be read and understood by someone who is at that grade level or above. The Gunning Fog Index is another readability test that is used to assess the difficulty of a written text. Like the Flesch-Kincaid grade level, it is often used by educators and professionals to assess the readability of educational materials and other types of written content [14]. The SMOG index is a readability formula that is used to assess the difficulty of a written text. The SMOG index is considered a reliable measure of readability, and it is often used in healthcare settings to assess the readability of patient education materials [14].

Statistical analyses were performed using IBM SPSS statistics version 24 (IBM Corp., Armonk, NY). The variables were analyzed using visual (histograms, probability plots) and analytical methods (Kolmogorov-Smirnov/Shapiro-Wilk test) to determine whether or not they were normally distributed. Descriptive analyses were presented using means and standard deviations (SD) for normally distributed variables and medians and interquartile range (IQR) for the non-normally distributed variables. In the comparison of data, the Student's t-test was used for normally distributed data, while the Mann-Whitney U test was used for data not normally distributed. A p-value <0.05 was considered statistically significant. Ethics committee approval was not obtained as the study did not involve either human or animal participants/subjects.

## Results

We examined a total of 120 websites with the aid of the search terms. After applying the exclusion criteria, 41 sites were included in the study (Table 1). The median DISCERN score for the evaluated websites was 47, and the median JAMA score was 3. As for the responses provided by ChatGPT, the median DISCERN score was found to be 55, while the median JAMA score was 0. Upon analyzing the responses of both ChatGPT and websites, it was determined that JAMA scores were significantly different (p<0.001), whereas DISCERN scores were similar (p=0.394) (Table 2). The websites yielded an average Flesch-Kincaid grade level of 8.04, an average Gunning Fog Index of 9.92, and an average SMOG index of 10.86. Upon analyzing ChatGPT's responses, the average Flesch-Kincaid grade level was found to be 10.68, the average Gunning Fog Index was 13.77, and the average SMOG index was 13.38. Upon comparing the Flesch-Kincaid Grade Level, Gunning Fog Index, and SMOG index results between the groups, a statistically significant difference was detected (p<0.001) (Table 3).

**Table 1:** Websites included in the analysis

Number	Website
1	https://www.webmd.com/back-pain/ss/slideshow-low-back-pain-overview
2	https://www.spine-health.com/conditions/lower-back-pain/lower-back-pain-symptoms-diagnosis-and-treatment
3	https://www.healthline.com/health/low-back-pain-acute
4	https://www.mayoclinic.org/diseases-conditions/back-pain/symptoms-causes/syc-20369906
5	https://www.nhs.uk/conditions/back-pain/
6	https://en.wikipedia.org/wiki/Low_back_pain
7	https://www.aans.org/en/Patients/Neurosurgical-Conditions-and-Treatments/Low-Back-Pain
8	https://www.hopkinsmedicine.org/health/conditions-and-diseases/back-pain/lower-back-pain-what-could-it-be
9	https://www.spineuniverse.com/conditions/low-back-pain
10	https://my.clevelandclinic.org/health/diseases/7936-lower-back-pain
11	https://www.hopkinsmedicine.org/health/conditions-and-diseases/back-pain/low-back-pain
12	https://www.healthline.com/health/back-pain
13	https://www.uwmedicine.org/conditions-symptoms/bone-joint-muscle/lower-back-pain
14	https://orthoinfo.aaos.org/en/diseases--conditions/meniscus-tears/
15	https://www.physio-pedia.com/Meniscal_Lesions
16	https://www.bupa.co.uk/health-information/knee-pain/meniscal-tear
17	https://www.orthobullets.com/knee-and-sports/3005/meniscal-tears
18	https://www.webmd.com/pain-management/knee-pain/meniscus-tear-injury
19	https://www.hopkinsmedicine.org/health/conditions-and-diseases/torn-meniscus
20	https://www.hss.edu/condition-list_torn-meniscus.asp
21	https://www.uptodate.com/contents/meniscal-injury-of-the-knee
22	https://www.medicalnewstoday.com/articles/meniscus-injury
23	https://www.mayoclinic.org/diseases-conditions/torn-meniscus/symptoms-causes/syc-20354818
24	https://www.healthline.com/health/osteoarthritis/knee-pain/meniscus-tear-recovery-time-without-surgery#when-to-see-a-doctor
25	https://www.nhs.uk/conditions/meniscus-tear/
26	https://bestpractice.bmj.com/topics/en-gb/826
27	https://cbphysiotherapy.in/condition/meniscal-injury
28	https://patient.info/bones-joints-muscles/sports-injuries/meniscal-tears-knee-cartilage-injuries
29	https://emedicine.medscape.com/article/308054-overview
30	https://orthoinfo.aaos.org/en/diseases--conditions/arthritis-of-the-knee/
31	https://www.hopkinsmedicine.org/health/conditions-and-diseases/knee-arthritis
32	https://my.clevelandclinic.org/health/diseases/21978-arthritis-of-the-knee
33	https://www.versusarthritis.org/about-arthritis/conditions/osteoarthritis-of-the-knee/
34	https://www.nhs.uk/conditions/osteoarthritis/
35	https://www.webmd.com/osteoarthritis/ostearthritis-of-the-knee-degenerative-arthritis-of-the-knee
36	https://www.hss.edu/condition-list_knee-arthritis.asp
37	https://www.healthline.com/health/osteoarthritis/knee-arthritis-symptoms
38	https://sportsmedicine.mayoclinic.org/condition/kneearthritis/
39	https://en.wikipedia.org/wiki/Knee_arthritis
40	https://www.verywellhealth.com/knee-arthritis-2548572
41	https://www.arthritis.org/diseases/more-about/osteoarthritis-of-the-knee

**Table 2:** DISCERN and JAMA scores of the groups *Statistically significant IQR: interquartile range; JAMA: Journal of the American Medical Association

	Websites, median (IQR)	ChatGPT, median (IQR)	P-value
DISCERN score	47 (22)	55 (4)	0.394
JAMA score	3 (1)	0 (0)	<0.001*

**Table 3:** Flesch-Kincaid grade level, Gunning Fog Index, and SMOG Index of the groups *Statistically significant SD: standard deviation; SMOG: Simple Measure of Gobbledygook

	Websites, mean ±SD	ChatGPT, mean ±SD	P-value
Flesch-Kincaid grade level	8.04 ±1.49	10.68 ±1.32	<0.001*
Gunning Fog Index	9.92 ±1.79	13.77 ±1.32	<0.001*
SMOG Index	10.86 ±1.24	13.38 ±0.94	<0.001*

## Discussion

The emergence of AI will surely usher in innovations in many walks of human life, including the field of health, and even our daily activities and habits may be influenced by AI over time. ChatGPT, an AI chatbot, possesses the ability to source data from various sources on the internet and employs natural language processing to generate human-like conversational dialogue [15]. Its ability to answer questions rapidly provides a great convenience in accessing the data on the internet. The questions we addressed in this study were as follows: (1) How efficient is this new technology in accessing the correct information? (2) How valuable is the information obtained? In our study, the quality and readability of the information obtained from ChatGPT were compared with those provided by health-related websites. Based on our findings, ChatGPT provides information that is similar in quality to the websites examined in the study. However, the information it provided was insufficient in terms of aspects that make up the JAMA benchmark criteria: (1) identification of authorship, (2) identification of sources, (3) specifying the date of creation/update, and (4) disclosures (of ownership, advertising policy, sponsorship, and conflicts of interests). In addition, when comparing ChatGPT and websites in terms of readability, It was observed that the data provided by ChatGPT were significantly more difficult to read than the information on the websites (p<0.001).

ChatGPT is the most advanced chatbot ever created. Its ability to give logical answers to questions within seconds and create texts that are indistinguishable from texts written by humans brings to mind the theories of the Day of Judgment [16]. In addition, its potential to be used in online exams or in areas such as scientific research raises serious concerns. However, although it is an advanced AI product, this chatbot has weaknesses as well as strengths when compared to websites.

This technology understands many languages. The fact that it can respond appropriately to different language inputs facilitates access to information. Given that a significant number of websites in the field of health are in English, which is considered a universal language, it makes accessing information relatively easy for those who do not speak English [17]. This quality of providing human-like responses is a major strength of the application [18]. In addition, ChatGPT responds within seconds to the queries put to it [16].

However, this technology has some weaknesses as well. It may not understand complex questions and may misunderstand words or phrases that it has not seen before. As a result, it may give wrong answers to such questions. The answer provided may still appear reasonable to the user and the user may perceive the incorrect information as correct. This chatbot, which finds sources on its own, does not allow the user to select the information source. The wrong information provided by ChatGPT can cause major issues, especially in the legal and medical fields [19-21]. ChatGPT provides information on data up to September 2021. Currently, it is not possible to obtain up-to-date and real-time data [18,19]. This is a big disadvantage compared to websites, especially at a time when medicine is developing rapidly.

In our study, there was no significant difference between ChatGPT and websites in terms of the DISCERN score. We attribute this to the fact that the questions put to ChatGPT included simple and high-search-volume terms. According to JAMA benchmark criteria, ChatGPT received 0 points in all searches with regard to information transparency and reliability. This makes the data source unreliable according to these criteria. When the medical information obtained was compared in terms of readability, ChatGPT was found to be more difficult in terms of readability in all three tests. In these tests, many factors such as the length of the sentences, the use of complex words, and the total syllable count are evaluated. In our study, information about health on websites was found to be more easily readable.

This study has a few limitations. Google search results may vary based on the region/country that the search originates from, although we deleted the browser history. In our study, five different answers were generated by means of the "regenerate response" option; since no similar study on this topic has been conducted before, we are unaware if this number will hold true in all cases. We recommend further studies in various other health-related fields and involving larger databases to gain deeper insights into the strengths and weaknesses of ChatGPT.

## Conclusions

AI models are technologically advanced products, with significant potential to improve our lives in several areas. They are immensely adaptable to change and progressive advancements. Notably, the integration of AI models into chatbots may give the impression that users receive specialist knowledge. Nevertheless, ChatGPT, an AI product, presently lags behind health-related websites in terms of providing reliable information of merit. Nevertheless, we envisage that these nascent AI-assisted conversational tools will continue to advance to the point where they become potent means for obtaining precise, lucid, and reliable information in the foreseeable future.
